# Semi-Interpenetrating Polymer Networks Based on Hydroxy-Ethyl Methacrylate and Poly(4-vinylpyridine)/Polybetaines, as Supports for Sorption and Release of Tetracycline

**DOI:** 10.3390/polym15030490

**Published:** 2023-01-17

**Authors:** Aurica Ionela Gugoasa, Stefania Racovita, Silvia Vasiliu, Marcel Popa

**Affiliations:** 1 Departament of Natural and Synthetic Polymers, Faculty of Chemical Engineering and Environmental Protection, “Gheorghe Asahi” Technical University of Iasi, Prof. Dr. Docent Dimitrie Mangeron Street, No. 73, 700050 Iasi, Romania; 2 “Petru Poni” Institute of Macromolecular Chemistry, Grigore Ghica Voda Alley, No. 41 A, 700487 Iasi, Romania; 3 Academy of Romanian Scientists, Ilfov Str., Nr. 3, Sector 5, 050044 Bucuresti, Romania

**Keywords:** semi-interpenetrating network, polybetaines, hydroxyethyl methacrylate, tetracycline, antimicrobial activity

## Abstract

Semi-interpenetrating polymer networks (semi-IPN) represent a type of polymeric material that has gained increasing amount of interest for their potential biomedical application. This study presents the synthesis, characterization and tetracycline loading/release capacities of semi-IPNs based on hydroxyethyl methacrylate (HEMA) and poly(4-vinylpyridine) (P4VP) or poly (1-vinyl-4-(1-carboxymethyl) pyridinium betaine) (P4VPB-1) and poly (1-vinyl-4-(2-carboxyethyl) pyridinium betaine) (P4VPB-2). The optimization of the semi-IPNs synthesis was achieved by studying the influence of reaction parameters (chemical structure of the cross-linking agent, HEMA:crosslinker ratio, HEMA:linear polymers ratio and the type of solvent of the linear polymers) on the yield of obtaining semi-IPNs and swelling capacity of these systems. Fourier-transform infrared analysis and scanning electron microscopy highlighted the chemical structures and morphologies of the semi-IPNs. The higher swelling capacity was observed in the case of the PHEMA/P4VPB-2 network due to the increased hydrophilicity of P4VPB-2 compared with P4VP and P4VPB-1 polymers. In vitro release studies of tetracycline reveal that the release mechanism is represented by non-Fickian diffusion being controlled by both diffusion and swelling processes. The antimicrobial activity of semi-IPN–tetracycline systems was tested against *E. coli* and *S. aureus*, demonstrating that tetracycline is released from the semi-IPN and retains its bactericidal activity. An increased value of the inhibition zone diameter compared with that of tetracycline indicates the possibility that the semi-IPN containing P4VPB-2 also exhibits intrinsic antimicrobial activity due to the presence of the polybetaine in the network structure.

## 1. Introduction

Hydrogels are chemically or physically crosslinked networks that can be obtained from natural or synthetic substances and are able to retain large amounts of water and biological fluids [1,2,3]. Hydrogels can be classified in function of various criteria, such as source (natural, synthetic, hybrid hydrogels) [4,5,6], physical properties [7], nature of cross-linking (physically and chemically crosslinked hydrogels) [8,9], type of ionic charges (anionic, cationic, zwitterionic hydrogels) [10,11,12], structure of the network (amorphous, semicrystalline, supramolecular, hydrocolloid aggregates) [13,14], size of pores (nonporous, micro-, meso- and macroporous hydrogels) [15], chemical structure of the network (homopolymeric, copolymeric, semi-IPN and IPN networks) [16,17,18] and biodegradability (non-biodegradable and biodegradable hydrogels) [19].

Due to their specific properties the hydrogels have many applications in biomedical and pharmaceutical fields, biotechnology or in separation and water purification processes [2].

Among the hydrogels, the semi-IPNs have gained particular interest for medical use due to their special properties.

1. Their porous structure with interconnected pores and high specific surface area leading to an increased amount of immobilized drug and a controlled release profile [20].
2. The improvement of their mechanical properties through introduction of a linear polymer into the three-dimensional network, compared with the classical hydrogel. The linear polymer can interact with the three-dimensional network through physical bonds such as: electrostatic interactions, hydrogen bonds, Van der Waals interactions, hydrophobic interactions or combinations thereof [21].
3. The ability to combine the hydrophobic and hydrophilic polymers to shape the properties of the hydrogel [22].
4. Their sensitivity to various external stimuli, obtaining so-called smart or intelligent hydrogels [23,24,25,26,27].

Hydroxyethyl methacrylate is one of the most widely used monomers in biomedical applications and poly(hydroxyethyl methacrylate) (PHEMA) is well known for its lack of toxicity, its biocompatibility, its high resistance to degradation and its hydrolysis under physiological conditions. Polymers based on HEMA were first mentioned in a study from 1936 [2], but only after 1960 when Wichterle and Lim [28] obtained the first hydrogel based on HEMA, was a way opened for the preparation of new types of hydrogels with various chemical structures being designated for use in biomedical applications. In recent years, semi-IPNs consisting of crosslinked networks based on HEMA and linear polymers that can be natural (alginate, selacan, chitosan, hyaluronic acid, chondroitin sulphate) or synthetic (poly(vinyl alcohol), poly(N-vinylpyrrolidone)) have been prepared and used in different applications [29,30,31,32,33]. Regarding the semi-IPNs based on HEMA and another synthetic polymer, the information is quite limited. For example, the semi-IPNs based on PHEMA and poly(N-vinylpyrroplidone) with suitable properties were synthesized by radical polymerization in order to be used for the cleaning of artistic surfaces or water-sensitive cultural heritage artifacts [33,34].

In this study, the choice of monomers (HEMA, dimethacrylic monomers) and linear polymers (P4VP and polybetaines (PB)) was based on the desire to develop polymeric materials with novel architectures that possess the ability to release the drug (tetracycline) in a controlled/sustained manner for use in dental applications, particularly in the treatment of periodontal disease. The benefits of using PHEMA in the biomedical field have been presented above and the choice of polybetaines as linear polymers was based on the fact that these synthetic polymers are known to be polymers with antifouling and antimicrobial properties [35]. Their incorporation into the polymer network may lead to new types of polymeric materials that can be used alone or together with a drug as systems with synergistic antimicrobial activity, but also with controlled/sustained release of the biological active principle.

## 2. Materials and Methods

### 2.1. Materials

HEMA, supplied from Merk Company KGaA (Darmstadt, Germany), was purified by vacuum distillation immediately prior to use. Poly(4-vinylpiridine) (M_W_ = 60,000 g/mol), dimethacrylic monomers: ethylene glycol dimethacrylate (EGDMA), diethylene glycol dimethacrylate (DEGDMA) and triethylene glycol dimethacrylate (TEGDMA), N,N,N′N′-tetramethylethylenediamine (TEMED), ammonium persulfate (APS), sodium bicarbonate, tetracycline (M = 444.43 g/mol), methanol and ethanol were purchased from Sigma-Aldrich and were used as received.

**Scheme 1:**
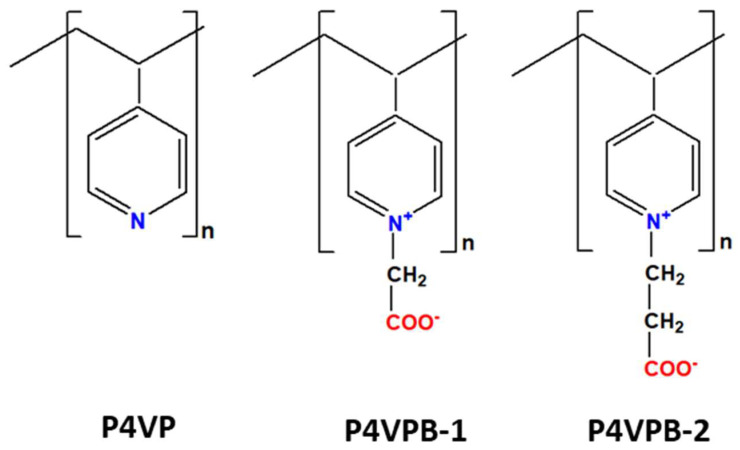
The chemical structures of synthetic polymer used in the preparation of semi-IPN.

The synthesis of poly(betaines) derived from P4VP has already been presented in other works [36,37]. Poly(betaines) were obtained via polymer-analogous reactions, the betainization agents being sodium monochloroacetate (P4VPB-1) and acrylic acid (P4VPB-2). The chemical structures of P4VP, P4VPB-1 and P4VPB-2 are presented in Scheme 1. Polybetaines were obtained with different degrees of betainization, as follows: 95% for P4VPB-1 and 96% for P4VPB-2.

### 2.2. Synthesis of Semi-Interpenetrating Polymer Networks

PHEMA/P4VP, PHEMA/P4VPB-1 and PHEMA/P4VPB-2 semi-IPNs were synthesized under the following conditions:

- initial monomer concentration (HEMA + dimethacrylic monomers) of C_0_ = 10% was kept constant for all experiments;
- different molar ratios of the crosslinkers (EGDMA, DEGDMA or TEGDMA) to the monomer (HEMA) were as follows 1:20; 1:40; 1:50; 1:67 and 1:80 (mol/mol);
- different gravimetric ratios of HEMA to the linear polymers (P4VP or PB) were as follows 1:0.2; 1:0.3; 1:0.4; 1:0.5 (g/g);
- the redox initiator concentration was C_i_ = 2 g (APS + TEMED)/100 g monomers.

For example, to obtain semi-IPNs with C_0_ = 10%, EGDMA:HEMA = 1:40 (mol/mol) and HEMA:P4VP or PB = 1:0.2 (g/g) was used: 7.37 mmol HEMA, 0.187 mmol EGDMA, 1 mL APS 2%, 1 mL TEMED 2%, and 0.193 g P4VP or PB dissolved in 7 mL solution of different solvents. The following combinations were chosen as solvents for dissolving the linear polymers: sodium bicarbonate; methanol:sodium bicarbonate (40:60, *v*/*v*); and ethanol:sodium bicarbonate (40:60, *v*/*v*). The solution obtained by mixing the components mentioned above was bubbled with nitrogen to remove the oxygen and was then transferred into a 10-mL syringe which was sealed and immersed in a thermostated bath at T = 50 °C for 8 h. After polymerization, the semi-IPNs were purified by washing with the used solvents and then with water to remove homopolymers and other unreacted substances. Finally, the semi-IPNs were cut into pieces of about 10 mm and subsequently dried by lyophilization. For comparison, three-dimensional networks based on HEMA and the three dimethacrylic monomers were also prepared under the same conditions.

The yield of the semi-IPN (*η*) with three-dimensional PHEMA and semi-IPNs based on PHEMA was calculated using the following relation:

(1) $$\eta \left(\%\right)=\frac{M_{d}}{M_{r}}\cdot 100$$

where: *M_d_*—the mass of the reaction product in the dry state (g), and *M_r_*—amount of the reaction participants (g).

### 2.3. Physico-Chemical Characterization of Semi-IPNs

FT-IR spectra were recorded by KBr pellets technique using a Bruker Vertex 70 FT-IR spectrometer (Bruker, Ettlingen, Germany) collecting 124-scan with a resolution of 2 cm^−1^ in the frequency range of 400–4000 cm^−1^.

**Figure 1:**
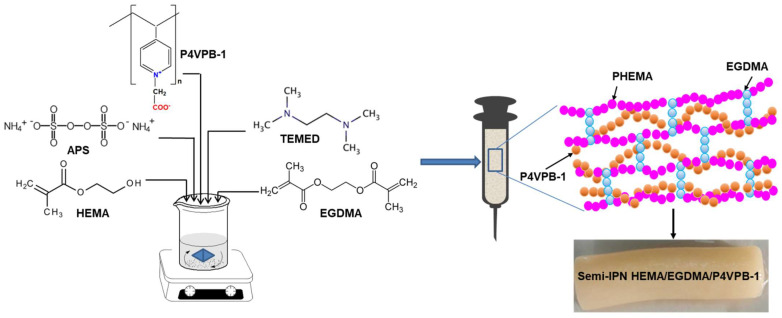
Graphical representation of synthesis reaction of semi-IPN based on HEMA, EGDMA and P4VPB-1.

In this study, the surface morphology of semi-IPNs was observed with a Quanta 200-FEI environmental scanning electron microscope coupled with an energy dispersive X-ray system (Hillsboro, OR, USA). Optical images presented in Table 1, Figure 1 and Figure 5 were realized with a DSLR Cannon EDS 4000D, 18MP, 18–55 mm SEE. The percentage of nitrogen in the semi-IPNs was determined by the Kjeldahl method [38].

Swelling studies were carried out using the gravimetric method. To determine the swelling capacity in water, the semi-IPNs were dried in air for 24 h and in vacuum at 40 °C for 48 h. The dried semi-IPNs (0.05 g) accurately weighed on the analytical balance were immersed in water at room temperature. At predetermined time intervals the swollen samples were removed from water and the surface was blotted with filter paper. Finally, the samples were weighed. The swelling measurements were performed until the equilibrium was reached and were run in triplicate. The swelling degree (*S_w_*) was obtained using the following equation:

(2) $$S_{w}\left(\%\right)=\frac{M_{e}-M_{0}}{M_{0}}\cdot 100$$

were *M_e_* is the weight of a swollen sample at equilibrium and *M*_0_ is the dried initial sample weight.

### 2.4. Sorption Batch Experiments

The studies of tetracycline immobilization onto semi-IPNs were carried out in a static system as follows. An amount of 0.1 g of semi-IPN with a known moisture was placed in Erlenmeyer flasks. Over the semi-IPN, 10 mL tetracycline solutions with concentrations between 0.25 and 1 mg/mL were added under gentle stirring (160 rpm., Thermostated Shaker Bath, Memmert, M00/M01, Schwabach, Germany) at different temperatures (298, 308 and 313 K) for different time intervals (10–100 min). After the specified period of time, the semi-IPN was removed quantitatively from the tetracycline solution by centrifugation at 1000 rpm for 10 min. The concentration of tetracycline in the supernatant solution before and after adsorption was determined using a UV–VIS Spectrophotometer (UV–VIS SPEKOL 1300, Analytik Jena, Jena, Germany) at a wavelength of 276 nm using a previously plotted calibration curve. The quantities of tetracycline adsorbed at any time, *q_t_* (mg/g) and at equilibrium, *q_e_* (mg/g) were calculated as follow:

(3) $$q_{t}=\frac{C_{01}-C_{t}}{W}\cdot V$$

(4) $$q_{e}=\frac{C_{01}-C_{e}}{W}\cdot V$$

where: *C*_01_—initial concentration of tetracycline solution (mg/mL); *C_t_*–concentration of tetracycline at any time (mg/mL); *C_e_*—concentration of tetracycline at equilibrium (mg/mL); *V*—volume of drug solution (mL); and *W*—amount of semi-IPNs (g).

### 2.5. In Vitro Release Studies

The release characteristics of the optimized systems were realized by incubation of 100 mg of drug–semi-IPN systems in 100 mL of dissolution media with pH = 1.2 (simulated gastric solution) for 2 h and pH = 7.4 (phosphate buffer solution) for 6 h at 37 °C, under gentle stirring (50 rpm). At predetermined time intervals, the dissolution medium was collected with microsyringes (1 μL) and the amount of tetracycline was determined spectrophotometrically (Nanodrop ND100, Wilmington, DE, USA) at the wavelengths of 269 nm (pH = 1.2) and 362 nm (pH = 7.4), respectively. The amount of tetracycline released was calculated using appropriate calibration curves.

### 2.6. Antimicrobial Activity Tests

Antimicrobial activity of tetracycline–semi-IPN systems was performed on two types of bacterial strains, namely: *Staphylococcus aureus* ATCC 25,923 (Gram positive bacteria) and *Escherichia coli* ATCC 25,922 (Gram negative bacteria) using the standard Kirby–Bauer disk-diffusion susceptibility method [39] according to the National Committee for Clinical Laboratory Standards Institute NCCLS, Standard Document M2-A5, Vilanova, PA, USA (2000). The microbial cultures were first regenerated from the storage medium and were prepared according to the manufacturer’s recommendations using suspensions in saline solution with a concentration of 5.2 × 10^7^ CFU/mL and density of 0.5 McFarland determined with Densimat Photometer. Antimicrobial tests were performed on tetracycline alone (micro-tablets of 30 μg tetracycline/disc) and tetracycline–semi-IPN systems (10-mm thick discs) which were deposited on bacterial cultures spread on Muller–Hinton agar in Petri dishes. After incubation at 37 °C for 24 h, the antimicrobial activity was monitored as the zone of inhibition being expressed as the average diameters of the inhibition zone measured in mm.

## 3. Results and Discussion

### 3.1. Synthesis and Optimized Semi-IPNs

PHEMA network and semi-IPNs were obtained by radical cross-linking reaction of HEMA with three dimethacrylic cross-linking agents in the absence or presence of linear polymers (P4VP, P4VPB-1 and P4VPB-2) using a redox system consisting of APS and TEMED as initiator. In cold or by mild heating conditions, the redox system provides several types of radicals, namely the alkylaminoethyl radical derived from TEMED and sulphate and hydroxyl radicals derived from the decomposition of APS (initiation stage) [40]. In the next step (propagation), the radicals formed in the initiation step react with the HEMA or with the dimethacrylic monomers (EGDMA or DEGDMA or TEGDMA), which act as cross-linking agents, leading to the formation of a growing macroradical and finally to a three-dimensional network. The termination step can occur through recombination reactions, disproportionation, chain transfer or inactivation of macroradicals by inhibitors.

As an example, the reaction to obtain the semi-IPN based on HEMA, EGDMA and P4VPB-1 is shown in Figure 1.

In case of semi-IPNs, poly(4-vinylpyridine) and polybetaines with one and two -CH_2_- groups between the ionic groups are retained within the three-dimensional PHEMA-based network by physical interactions.

In order to select the macromolecular support with suitable properties for the immobilization of water-soluble drugs, the optimization of semi-IPN synthesis is very important and this process was carried out by studying the influence of reaction parameters on the yield of semi-IPN and on swelling degree. For comparison, three-dimensional networks based on HEMA and three dimethacrylate monomers were obtained under similar reaction conditions. The reaction parameters were as follows: the chemical structure of the cross-linking agent; the molar ratio between the crosslinker and HEMA; the gravimetric ratio between HEMA and linear polymers; and the solvents in which the cross-linking reactions take place, these being also solvents for the linear polymers.

**Figure 2:**
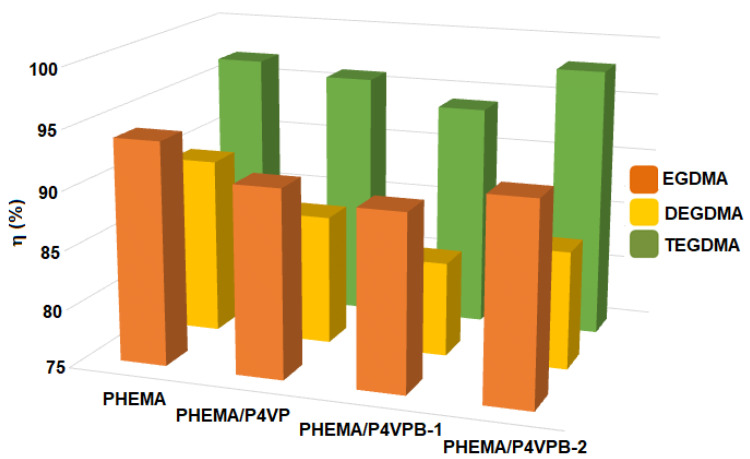
The influence of the chemical structure of the cross-linking agent on *η* (C_0_ = 10%, C_i_ = 2%, crosslinker: HEMA ratio = 1:20 (mol/mol), HEMA:linear polymer = 1:0.2 (g/g), T = 50 °C, t = 8 h, the solvent used to dissolve the synthetic polymers was ethanol:sodium bicarbonate (40:60, *v*/*v*).

**Figure 3:**
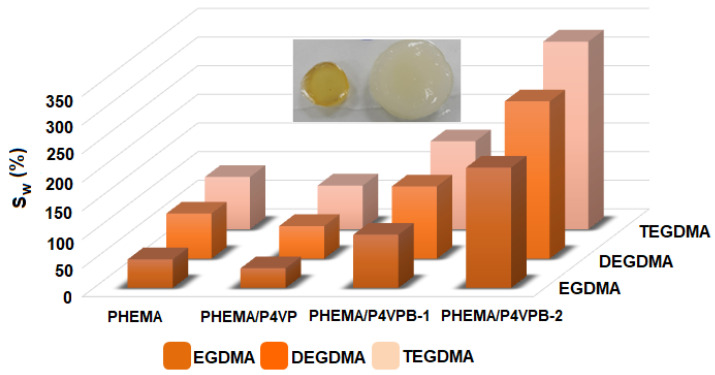
Influence of the chemical structure of the cross-linking agent on the maximum swelling degree in water.

Three cross-linking agents with similar structures but with different lengths of alkyl chain between the two methacrylic groups (EGDMA, DEGDMA, TEGDMA) were chosen to study the influence of the chemical structure of the cross-linking agent on η values (Figure 2) as well as on the swelling degree (Figure 3). The choice of these cross-linkers was made while taking into account that EGDMA, DEGDMA and TEGDMA are often used as cross-linking agents in the preparation of various polymeric materials with biomedical applications. Additionally, TEGDMA is well known as a component of the structure of dental resins [41].

The values of *η* for PHEMA and semi-IPNs shown in Figure 2 are in the range of 83 to 98% and are influenced by the chemical structure of the cross-linking agent as follows: *η_TEGDMA_* > *η_EGDMA_* > *η_DEGDMA_*. Thus, semi-IPNs with high values of *η* were obtained when the cross-linking agent was TEGDMA. The high values of *η* suggest that the monomers interacted almost completely, confirming that the cross-linking copolymerization reactions occurred. Additionally, from the values of *η* of the semi-IPNs, it can be concluded that a large part of the linear polymer introduced in the reaction remained within the semi-IPNs.

Figure 3 shows that the maximum degree of swelling has an increasing trend with increasing alkyl chain between the methacrylic groups of the cross-linker. As expected, the cross-linking agent with the longer chain between the two methacrylic groups led to a network with longer cross-linking bridges, thus with a less compact structure as well as with the possibility to adsorb a higher amount of water.

The swelling degree is also influenced by the chemical structure of the linear polymer. The introduction of polybetaines in the composition of semi-IPNs had a positive effect on the maximum swelling degree in water. The lower swelling degree of PHEMA/PVP and PHEMA/P4VPB-1 networks compared with PHEMA/P4VPB-2 networks are due to the fact that the P4VP and P4VPB-1 are hydrophobic and slightly hydrophilic, respectively, these polymers being insoluble in pure water. It is known from the literature that the hydrophilicity of polybetaines increases with increasing alkyl chain between the anionic and cationic groups [36]. For this reason, the PHEMA/P4VPB-2 networks are more hydrophilic and absorb more water molecules.

**Figure 4:**
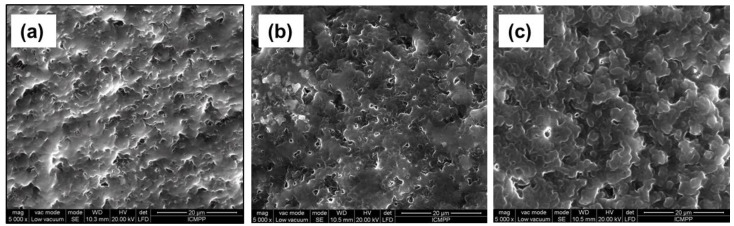
Influence of the cross-linker on the surface morphology of PHEMA/P4VP: (**a**) EGDMA; (**b**) DEGDMA; (**c**) TEGDMA, (solvent of P4VP = ethanol:sodium bicarbonate (40:60, *v*/*v*), HEMA:crosslinker = 1:50, mol/mol, HEMA:P4VP = 1:0.2 g/g).

SEM images were additionally performed to highlight the influence of the chemical structure of the cross-linking agent on the surface morphologies of semi-IPNs. The SEM images from Figure 4 show the influence of the cross-linking agents on the morphologies of PHEMA/P4VP semi-IPNs.

The networks with TEGDMA (Figure 4c) as cross-linker have a less compact structure with interconnected pores and larger pore sizes. This is probably due to the chemical structure of TEGDMA, which has a higher distance between the methacrylic groups leading to longer cross-linking bridges and larger network mesh sizes. The less compact network structure with TEGDMA is also confirmed by its swelling behavior (Figure 3).

To study the influence of the molar ratio between cross-linking agent and HEMA, the semi-IPN containing TEGDMA as cross-linker was chosen because this presents the highest values of both the yield of semi-IPN as well as of the swelling degree.

Consequently, the influence of the molar ratio between TEGDMA and HEMA was determined by modifying this parameter as follows: 1:80, 1:67, 1:50, 1:40 and 1:20 (mol/mol), and by keeping other parameters—the monomer and initiator concentrations (C_0_ = 10%, C_i_ = 2%), gravimetric ratio between HEMA and linear polymer (1:0.2 g/g), temperature (T = 50 °C), and reaction time (t = 8 h)—constant. The solvent used for linear polymers was ethanol:sodium bicarbonate (40:60, *v*/*v*).

By changing the molar ratio between TEGDMA and HEMA from 1:20 to 1:80 an increase of the values of maximum swelling degree was obtained. This behavior is probably due to the change in cross-link density. Thus, increasing the number of HEMA moles leads to the synthesis of networks with a lower degree of cross-linking and a higher swelling capacity in water, but with a low mechanical strength. For example, semi-IPNs with a high HEMA content are easily deformed. The data obtained for *η* and maximum swelling degrees in water are presented in Table 1.

Depending on the molar ratio between HEMA and TEGDMA and the type of synthetic polymer used, networks with a more compact structure and different colors are obtained: PHEMA networks—white color, PHEMA/P4VP—cream color, PHEMA/P4VPB-1—beige color and PHEMA/P4VPB-2—pink color. Additionally, the *η* presents high values ranging from 85 to 98%. For the following studies the ratio of 1:50 mol/mol between TEGDMA and HEMA was considered.

The gravimetric ratio HEMA:linear polymers is another factor influencing the structure of the semi-IPN and hence its properties. For the study of this parameter we considered the semi-IPN based on PHEMA and P4VPB-2 characterized by: TEGDMA:HEMA ratio = 1:50 (mol/mol); C_0_ = 10%, C_i_ = 2%; solvent used for linear polymers was ethanol: sodium bicarbonate (40:60, *v*/*v*); and HEMA:linear polymer = 1:0.2; 1:0.3; 1:0.4; 1:0.5 g/g; T = 50 °C, t = 8 h.

**Figure 5:**
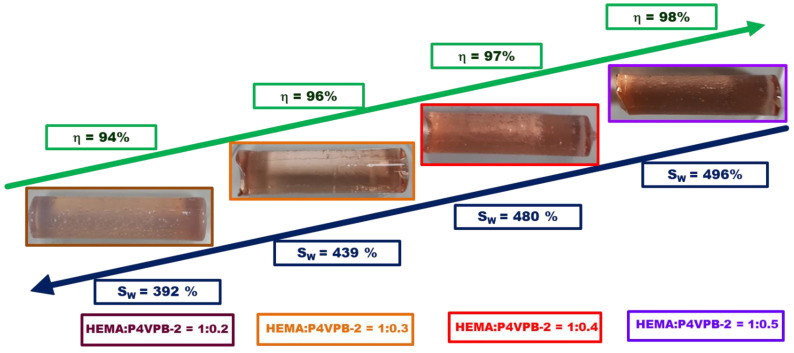
Optical images of HEMA/P4VPB-2 semi-IPNs prepared with various gravimetric ratios between HEMA and P4VPB-2.

By using different ratios between HEMA and linear polymer, the color of the semi-IPNs was changed, as shown in Figure 5.

The color of the semi-IPN is probably due to the presence of linear polymers which have different colors in their powder forms as follows: P4VP—dirty white; P4VPB-1—light green; and P4VPB-2—green. Color can also result from the physical interactions that are established between linear polymers and the polyHEMA network. It should be mentioned that the cross-linked networks based on HEMA are white. By analyzing the appearance of the semi-IPNs and by correlating the values of η as well as of maximum swelling degree with the amount of P4VPB-2 retained in the polymer network structure (Table 2), the following comments can be made:

- the color of PHEMA/P4VPB-2 semi-IPNs changes from pale pink to reddish brown, a sign that polybetaine is retained in higher amounts within the polymer networks when HEMA:P4VPB-2 is 1:0.5, g/g;
- *η* values increase slightly from 94% to 98% with increasing the amount of linear polymer retained;
- the amount of linear polymers (P4VP, P4VPB-1 and P4VPB-2) retained in the semi-IPN structure can be determined by the nitrogen content using the Kjeldahl method (Table 2) [38];
- the values of the maximum swelling degree increase with the increasing amount of polybetaine, because P4VPB-2 is a hydrophilic polymer and its retention in the structure of semi-IPN leads to the formation of polymeric materials with a high water swelling capacity.

The differences between the experimental and calculated values of nitrogen content appear due to the removal of small quantities of P4VPB-2 from the surface or surface layers in the purification process of semi-IPN, a similar phenomenon being encountered in other semi-IPNs.

**Figure 6:**
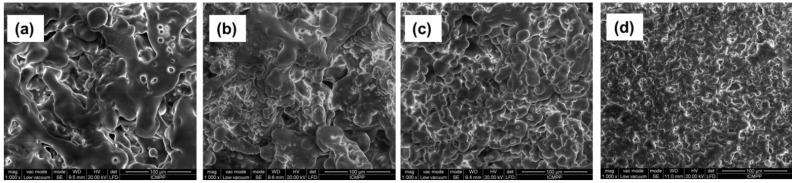
Influence of HEMA:P4VPB-1 ratio on the surface morphology of PHEMA/P4VPB-1 (**a**) 1:0.2, g/g; (**b**) 1:0.3, g/g; (**c**) 1:0.4, g/g; (**d**) 1:0.5,g/g (solvent of P4VPB-1 = ethanol:sodium bicarbonate (40:60, *v*/*v*), HEMA:TEGDMA = 1:50, mol/mol).

At different ratios of HEMA:linear polymer, semi-IPN networks with different morphologies and sponge-like structures with interconnected pores are obtained (Figure 6). Additionally, as the amount of polybetaine increases, the polymer network structure becomes more compact and homogeneous.

**Figure 7:**
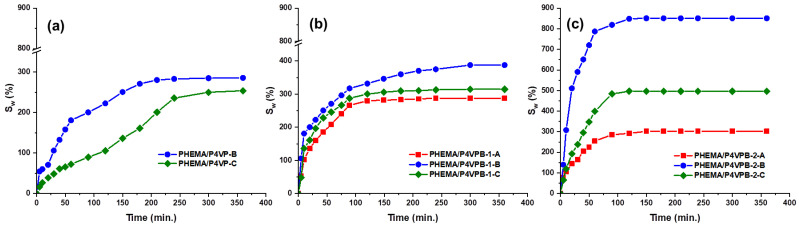
The influence of the solvents of linear polymers on the degree of swelling in water of PHEMA/P4VP (**a**), PHEMA/P4VP-1 (**b**) and PHEMA/P4VP-2 (**c**): A-NaHCO_3_ (0.25N); B-CH_3_OH:NaHCO_3_ (40:60, *v*/*v*); and C-C_2_H_5_OH:NaHCO_3_ (40:60, *v*/*v*).

Another important parameter influencing the swelling degree and the yield of semi-IPN is the solvent used for the linear polymers, i.e., the solvent in which the cross-linking reaction takes place. The solubility of the three linear polymers differs as follows: P4VP is soluble in alcohol, acetic acid, DMF, and toluene; P4VPB-1 is soluble in water with added salts; and P4VPB-2 is soluble in water and alcohols. For this reason, the following substances were chosen as reaction media and as solvents for the linear polymers: sodium bicarbonate, methanol:sodium bicarbonate (40:60, *v*/*v*), and ethanol:sodium bicarbonate (40:60, *v*/*v*) (Figure 7). The influence of the solvent was performed on the semi-IPNs with the following characteristics: TEGDMA:HEMA ratio = 1:50 (mol/mol); C_0_ = 10%, C_i_ = 2%; HEMA:linear polymer = 1:0.5 (g/g); T= 50 °C and t = 8 h.

Semi-IPNs with the highest degree of swelling in water are those in which the linear component is polybetaine with two -CH_2_- groups between the anionic and cationic groups. As mentioned above, among the linear polymers used, this polybetaine exhibits the highest hydrophilicity; therefore, by including it in the PHEMA network, a polymeric material with a higher capacity to absorb water molecules is obtained. The water swelling behavior of semi-IPNs is dictated by the nature of the solvent in which the cross-linking reaction takes place and hence that for dissolving linear polymers. This phenomenon can be explained by obtaining polymer networks with swelling degrees that can be influenced both by the behavior of the three polymers P4VP, P4VPB-1 and P4VPB-2 in solution and by the conformations adopted by them in the respective solvents.

**Figure 8:**
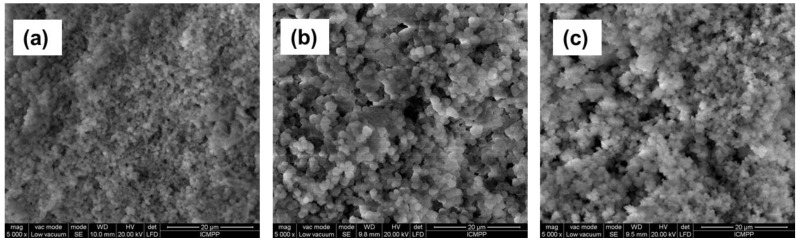
The influence of the solvent of P4VPB-2 on the surface morphology of PHEMA/P4VPB-2: (**a**) sodium bicarbonate, 0.25N; (**b**) CH_3_OH:sodium bicarbonate (40:60, *v*/*v*); (**c**) C_2_H_5_OH:sodium bicarbonate (40:60, *v*/*v*) (HEMA:TEGDMA = 1:50, mol/mol HEMA:P4VPB-2 = 1:0.5 g/g).

The nature of the solvent for the linear polymer has a strong influence on the morphology of the semi-IPN networks (Figure 8). For example, in the case of semi-IPNs based on PHEMA and P4VPB-2 it can be observed that polymer materials with a different pore structure are formed, probably due to the different behavior of P4VPB-2 in sodium bicarbonate solution or in the alcohol–sodium bicarbonate mixture.

**Figure 9:**
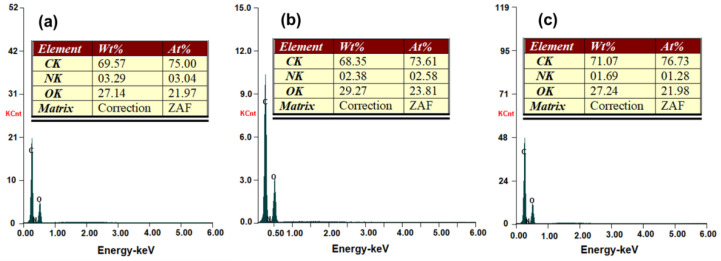
EDX analysis for PHEMA/P4VP (**a**), PHEMA/P4VPB-1 (**b**) and PHEMA/P4VPB-2 (**c**) semi-IPNs (TEGDMA:HEMA = 1:50, mol/mol; HEMA:linear polymers = 1:0.5 g/g, solvent of linear polymer = ethanol:sodium bicarbonate (40:60, *v*/*v*).

The presence of P4VP, P4VPB-1 and P4VPB-2 in the structure of the semi-IPNs was also confirmed by EDX analysis by the presence of the nitrogen atom on the surface of the analyzed samples (Figure 9).

**Figure 10:**
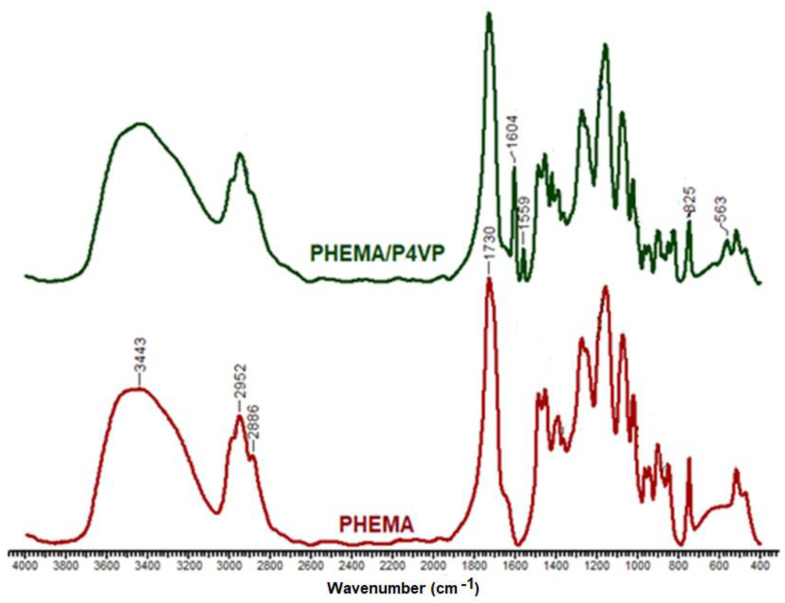
FT-IR spectra of PHEMA and semi-IPN based on PHEMA and P4VP.

**Figure 11:**
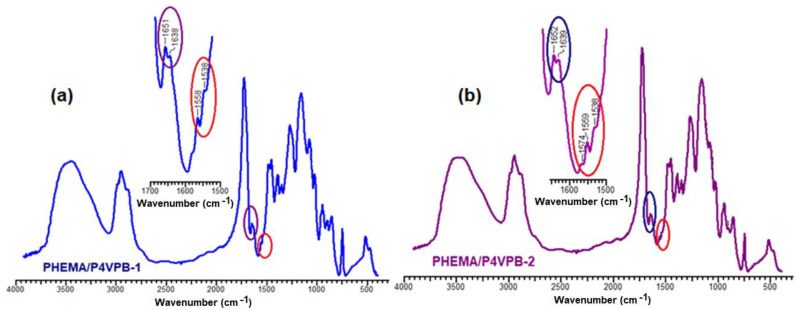
FT-IR spectra of PHEMA/PVPB-1 (**a**) and PHEMA/PVPB-2 (**b**) semi-IPNs.

The obtained semi-IPNs were investigated using FT-IR spectroscopy to provide information on their chemical structure as well as to prove that the monomer (HEMA) and the activator/initiator used in the polymerization reaction had been completely removed from the polymeric networks. In Figure 10 and Figure 11 are presented the FT-IR spectra of PHEMA, PHEMA/PVP, PHEMA/PVPB-1 and PHEMA/PVPB-2 semi-IPNs.

The characteristic PHEMA absorption bands are located at:

- 3443 cm^−1^, attributed to the valence vibration of OH (νO-H) groups involved in hydrogen bond formation;
- 2952 and 2886 cm^−1^, which are characteristic of the valence vibrations of the symmetric and asymmetric -CH_2_- group;
- 1730 cm^−1^, the stretching vibration of the >C=O bond belonging to the ester group located in the cross-linker molecule.

In the spectrum of the semi-IPN based on PHEMA and P4VP (Figure 10), new characteristic P4VP bands are observed, namely:

- 1604 and 1559 cm^−1^, attributed to the vibrations of the pyridine ring (C=C and C–N);
- 825 and 563 cm^−1^, specific to out-of-plane pyridine ring deformation vibrations.

For PHEMA/P4VPB-1 and PHEMA/P4VPB-2, the appearance of the pyridinium ion and carboxylate ion absorption bands at 1651 and 1538 cm^−1^ (PHEMA/P4VPB-1) (Figure 11a) and at 1652, 1574 and 1538 cm^−1^ (PHEMA/P4VPB-2) (Figure 11b) attest to the presence of both polybetaines in the structure of the semi-IPNs.

### 3.2. Sorption Studies of Tetracycline, Selection of Optimized Formulations

**Figure 12:**
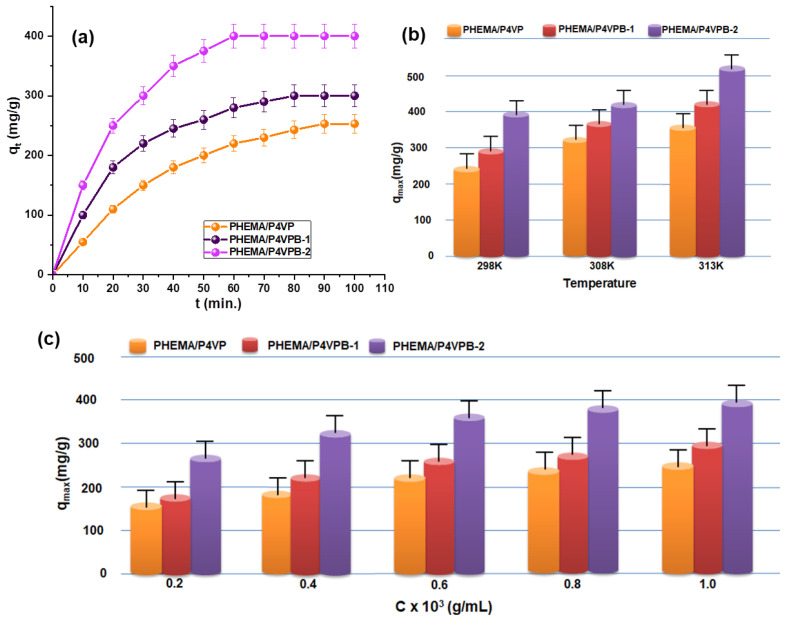
The influence of sorption parameters on the sorption capacity of semi-IPNs: (**a**) contact time (C_tetracycline_ = 1 mg/mL, T = 298 K); (**b**) temperature (C_tetracycline_ = 1 mg/mL, T = 298 K); (**c**) concentration of tetracycline solution (T = 298 K).

The influence of several parameters (contact time, initial drug concentration and temperature) on the sorption process of tetracycline onto semi-IPNs was evaluated in order to find the most suitable drug delivery system in terms of highest amount of tetracycline immobilized. The influence of sorption parameters is illustrated in Figure 12.

From Figure 12a we can observe that the contact time at equilibrium is 90 min for PHEMA/P4VP, 80 min for PHEMA/P4VPB-1 and 60 min for PHEMA/P4VPB-2. Once equilibrium is reached, the amount of drug adsorbed remains constant. PHEMA/P4VPB-2 networks show increased affinity for tetracycline compared to PHEMA/P4VP and PHEMA/P4VPB-1 networks. This behavior is based on the two properties of PHEMA/P4VPB-2 networks, namely the porous structure as seen in SEM images (Figure 8) as well as the higher hydrophilicity compared to PHEMA/P4VP and PHEMA/P4VPB-1, demonstrated by water swelling studies (Figure 7). Tetracycline is sorbed on semi-IPNs as follows: PHEMA/P4VP < PHEMA/P4VPB-1 < PHEMA/P4VPB-2 (Figure 12b,c). The sorption rate increases with increasing drug solution concentration and at high drug concentrations there is an increase in the number of interactions between semi-IPNs and sorbate ions. The adsorption of tetracycline on semi-IPNs is favored by high temperature, because the increase of this parameter leads to an increase in molecular motion as well as in the diffusion rate of the drug, facilitating the retention of the drug molecules in the pores more easily. Consequently, a greater amount of tetracycline is retained in the semi-IPNs at higher temperature (Figure 12b).

### 3.3. Release Studies of Tetracycline from Semi-IPN–Drug Systems

**Figure 13:**
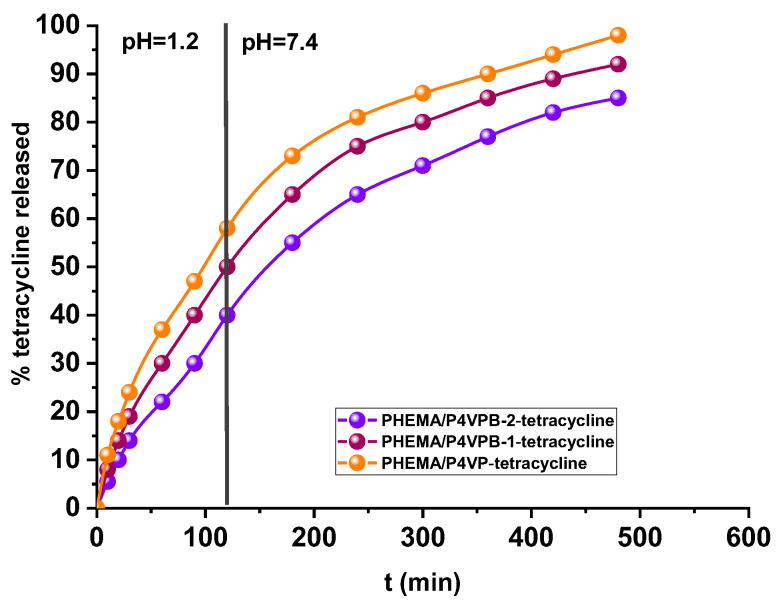
Release profile of tetracycline from semi-IPN–drug systems.

Tetracycline release studies from semi-IPN–drug systems were performed in buffer solutions at pH = 1.2 and pH = 7.4 and were carried out only for systems containing the highest amount of immobilized tetracycline. The amount of tetracycline released over time is shown in Figure 13 and the values of the kinetic parameters of the release process are shown in Table 3.

From the evaluation of the rate constants shown in Table 3, obtained by applying the first-order kinetic model, the Higuchi model and the Korsmeyer–Peppas model, it can be seen that the lowest release rate of tetracycline was obtained in the case of the PHEMA/P4VPB-2 network, probably due to stronger interactions between the drug molecule and the ionic groups of polybetaine, but also to the different behavior of PHEMA/P4VPB-2 in the release media compared with PHEMA/P4VP and PHEMA/P4VPB-1. The diffusional exponent, *n*, has values in the range of 0.659–0.761, indicating that the tetracycline release mechanism is characteristic of non-Fickian diffusion. Additionally, values of *n* < 0.85 indicate that semi-IPNs swell in the release media and do not undergo degradation or erosion processes. Thus, the mechanism of tetracycline release is controlled by the swelling process of semi-IPNs followed by the diffusion of the drug through the pores. The high values of R^2^ (0.996–0.999) obtained when applying the Higuchi and Korsmeyer–Peppas models show that these models describe the experimental data quite well.

### 3.4. Antimicrobial Activity of Tetracycline-Loaded Semi-IPNs

The antimicrobial activity of semi-IPNs with immobilized tetracycline was performed on two types of bacterial strains, namely: Staphylococcus aureus ATCC 25,923 (Gram positive bacteria) and Escherichia coli ATCC 25,922 (Gram negative bacteria). These strains were tested for their behavior towards tetracycline showing that they were sensitive to the drug. Antimicrobial tests were performed on tetracycline and semi-IPN drug-embedded systems in the form of 10-mm thick discs, which were deposited on bacterial cultures displayed on Mueller–Hinton agar in Petri dishes. Dishes were read after incubation for 18 and 24 h at 37 °C. The appearance of zones of inhibition of bacterial cultures, of variable size, around the test sample demonstrates that tetracycline is active and killed the bacteria. Bacterial culture inhibition zones are shown in Table 4.

The results of this test show that the antibiotic is released from semi-IPNs and retains its bactericidal capacity, as demonstrated by the appearance of bacterial culture inhibition zones around the products.

The questions of whether tetracycline fixed on semi-IPN networks is released in saline solution and whether it retains its bactericidal capacity were also investigated. Semi-IPN based on PHEMA and P4VPB-2 was chosen for this testing. Solutions of 0.1–0.2 mL saline were added over different amounts of PHEMA/P4VPB-2 and elution time was arbitrarily set to 20 min. Both resulting solution and semi-IPN were deposited on the spread bacterial cultures (*E. coli* and *S. aureus*), followed by incubation and plate reading after 18 and 24 h. Zones of inhibition with diameters ranging from 8 mm to 25 mm appeared, with the diameters of the inhibition zones increasing in direct proportion to the polymer/antibiotic ratio. The appearance of inhibition zones confirms that tetracycline had been released from the semi-IPNs and retained its bactericidal activity.

As a result of the studies performed it can be stated that the designed semi-IPN–tetracycline system was able to deliver the drug in a sustained manner under the specific pH conditions of the oral cavity (which can vary from weakly basic to acidic) and was able to exert antibacterial activity in the saline environment specific to saliva. Of course, studies are still needed on how to administer this system, but it can be anticipated that the introduction of particles obtained by finely grinding them or pipetting suspensions of them into the periodontal pocket, which can then be closed/sealed with specific adhesives currently used in dental practice, could be an alternative to the currently used classical treatment of periodontitis. Another option could be the creation of drug-loaded films that can then be implanted into the periodontal pocket.

## 4. Conclusions

A series of semi-IPNs were obtained by the radical cross-linking copolymerization reaction of HEMA with three dimethacrylic cross-linking agents (EGDMA, DEGDMA and TEGDMA) in the absence or presence of P4VP, P4VPB-1 and P4VPB-2, using a redox system consisting of APS and TEMED as initiators. The influence of some reaction parameters (chemical structure of cross-linkers, HEMA:crosslinker ratio, chemical structure of linear polymer, HEMA:linear polymer ratio, solvent of linear polymers) on the yield of semi-IPN and the swelling degree of the networks in water was studied. PHEMA and semi-IPNs were characterized by FT-IR spectroscopy and scanning electron microscopy. The presence of P4VP, P4VPB-1 and P4VPB-2 in the structure of semi-IPNs was also highlighted by determining the nitrogen content using the Kjeldahl method. An optimization study of the tetracycline sorption process on semi-IPNs was carried out. The drug release study showed that PHEMA/P4VPB-2 networks can be used as macromolecular supports to obtain sustained drug release systems and can be applied in the periodontal pocket to treat periodontitis. Antimicrobial testing of the polymer–drug system demonstrated that tetracycline is released from semi-IPN and retains its bactericidal activity. The semi-IPNs based on HEMA and the three linear polymers P4VP, P4VPB-1 and P4VPB-2) can be considered a candidate for the encapsulation or sorption of drugs with antimicrobial activity on the bacterial plaques in the oral cavity. These drug delivery systems can be administered either in the form of particle suspensions or in the form of films, representing viable and promising alternatives for the treatment of periodontal disease.

## Figures and Tables

**Table 1:** Values of *η*, maximum swelling degree in water and optical images of PHEMA, PHEMA/P4VP, PHEMA/P4VPB-1 and PHEMA/P4VPB-2 networks at different molar ratios HEMA: TEGDMA.

Sample Codes	TEGDMA:HEMA (mol/mol)	*η* (%)	*S_W_* (%)	Images of Semi-IPN
	1:80	90	285.7	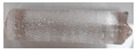
	1:67	92	270.9	-
PHEMA	1:50	93	222.5	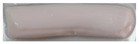
	1:40	98	200.8	-
	1:20	98	70.70	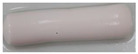
	1:80	85	242.7	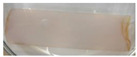
	1:67	87	226.3	-
PHEMA/P4VP	1:50	90	200.7	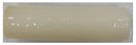
	1:40	96	138.6	-
	1:20	96	60.10	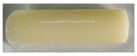
	1:80	88	386.91	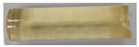
	1:67	89	330.16	-
PHEMA/P4VPB-1	1:50	91	246.26	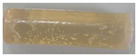
	1:40	94	153.70	-
	1:20	97	136.31	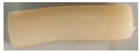
	1:80	91	550.72	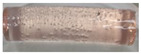
	1:67	93	472.54	-
PHEMA/P4VPB-2	1:50	94	391.60	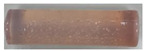
	1:40	97	327.61	-
	1:20	98	298.26	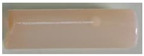

**Table 2:** Influence of the amount of P4VPB-2 on nitrogen content.

Sample Codes	TEGDMA/HEMA (mol/mol)	HEMA/P4VPB-2 (g/g)	N (%)		P4VPB-2 (%)
			Calc.	Exp.	Found in Semi-IPN
		1:0.2	1.292	1.204	93.19
P4VPB-2	1:50	1:0.3	1.795	1.710	95.26
		1:0.4	2.227	2.140	96.09
		1:0.5	2.603	2.534	97.35

**Table 3:** Kinetic parameters of tetracycline release process from semi-IPN–drug systems.

		PHEMA/P4VP	PHEMA/P4VPB-1	PHEMA/P4VPB-2
First-order kinetic model [42] *	*k*_1_ (h^−1^)	0.087	0.085	0.079
$logQ_{t}=logQ_{0}+\frac{k_{1}\cdot t}{2.303}$	R^2^	0.989	0.986	0.984
Higuchi model [43] *	*k_H_* (min^−0.5^)	4.813	4.426	3.831
$Q_{t}=k_{H}\cdot t^{1/2}$	R^2^	0.996	0.996	0.997
Korsmeyer–Peppas model [44] *	*k_r_* (min^−n^)	0.025	0.017	0.012
$M_{t}/M_{\infty }=k_{r}\cdot t^{n}$	*n*	0.659	0.725	0.761
	R^2^	0.998	0.999	0.999

* *Q_t_* = amount of drug released at time *t*; *Q*_0_ = initial amount of drug in solution; *k*_1_ = the first order rate constant; *k_H_* = Higuchi dissolution constant; *M_t_*/*M*_∞_ = fraction of drug released at time *t*; *k_r_* = release rate contant; *n* = diffusion coefficient.

**Table 4:** Diameters of inhibition zones.

Sample Codes	*E. coli* ATCC 25922	*S. aureus* ATCC 25923
PHEMA/P4VP-tetracycline	12 mm	20 mm
PHEMA/P4VPB-1-tetracycline	15 mm	23 mm
PHEMA/P4VPB-2-tetracycline	17 mm	31 mm
Tetracycline	20 mm	28 mm

## Data Availability

Not applicable.
